# Sexy ways: approaches to studying plant sex chromosomes

**DOI:** 10.1093/jxb/erae173

**Published:** 2024-04-23

**Authors:** Roman Hobza, Václav Bačovský, Radim Čegan, Lucie Horáková, Marcel Hubinský, Tomáš Janíček, Bohuslav Janoušek, Pavel Jedlička, Jana Kružlicová, Zdeněk Kubát, José Luis Rodríguez Lorenzo, Pavla Novotná, Vojtěch Hudzieczek

**Affiliations:** Department of Plant Developmental Genetics, Institute of Biophysics, Academy of Sciences of the Czech Republic, Kralovopolska 135, 612 00 Brno, Czech Republic; Department of Plant Developmental Genetics, Institute of Biophysics, Academy of Sciences of the Czech Republic, Kralovopolska 135, 612 00 Brno, Czech Republic; Department of Plant Developmental Genetics, Institute of Biophysics, Academy of Sciences of the Czech Republic, Kralovopolska 135, 612 00 Brno, Czech Republic; Department of Plant Developmental Genetics, Institute of Biophysics, Academy of Sciences of the Czech Republic, Kralovopolska 135, 612 00 Brno, Czech Republic; Faculty of Science, Masaryk University, Kamenice 5, 625 00 Brno, Czech Republic; Department of Plant Developmental Genetics, Institute of Biophysics, Academy of Sciences of the Czech Republic, Kralovopolska 135, 612 00 Brno, Czech Republic; Faculty of Science, Masaryk University, Kamenice 5, 625 00 Brno, Czech Republic; Department of Plant Developmental Genetics, Institute of Biophysics, Academy of Sciences of the Czech Republic, Kralovopolska 135, 612 00 Brno, Czech Republic; Faculty of Science, Masaryk University, Kamenice 5, 625 00 Brno, Czech Republic; Department of Plant Developmental Genetics, Institute of Biophysics, Academy of Sciences of the Czech Republic, Kralovopolska 135, 612 00 Brno, Czech Republic; Department of Plant Developmental Genetics, Institute of Biophysics, Academy of Sciences of the Czech Republic, Kralovopolska 135, 612 00 Brno, Czech Republic; Department of Plant Developmental Genetics, Institute of Biophysics, Academy of Sciences of the Czech Republic, Kralovopolska 135, 612 00 Brno, Czech Republic; Faculty of Science, Masaryk University, Kamenice 5, 625 00 Brno, Czech Republic; Department of Plant Developmental Genetics, Institute of Biophysics, Academy of Sciences of the Czech Republic, Kralovopolska 135, 612 00 Brno, Czech Republic; Department of Plant Developmental Genetics, Institute of Biophysics, Academy of Sciences of the Czech Republic, Kralovopolska 135, 612 00 Brno, Czech Republic; Department of Plant Developmental Genetics, Institute of Biophysics, Academy of Sciences of the Czech Republic, Kralovopolska 135, 612 00 Brno, Czech Republic; Faculty of Science, Masaryk University, Kamenice 5, 625 00 Brno, Czech Republic; Department of Plant Developmental Genetics, Institute of Biophysics, Academy of Sciences of the Czech Republic, Kralovopolska 135, 612 00 Brno, Czech Republic; University of South Bohemia in České Budějovice, Czech Republic

**Keywords:** Bioinformatics, chromosome dissection, cytogenetics, dioecious plants, epigenetics, functional genetics, sex chromosomes, tandem repeats, transposable elements

## Abstract

Sex chromosomes have evolved in many plant species with separate sexes. Current plant research is shifting from examining the structure of sex chromosomes to exploring their functional aspects. New studies are progressively unveiling the specific genetic and epigenetic mechanisms responsible for shaping distinct sexes in plants. While the fundamental methods of molecular biology and genomics are generally employed for the analysis of sex chromosomes, it is often necessary to modify classical procedures not only to simplify and expedite analyses but sometimes to make them possible at all. In this review, we demonstrate how, at the level of structural and functional genetics, cytogenetics, and bioinformatics, it is essential to adapt established procedures for sex chromosome analysis.

## Teaser

This review explores adapted or newly developed methods customized for comprehensive analysis of sex chromosomes in dioecious plants. We emphasize methodological approaches for understanding XY chromosome structure and function.

## Introduction

**Fig. 1.**
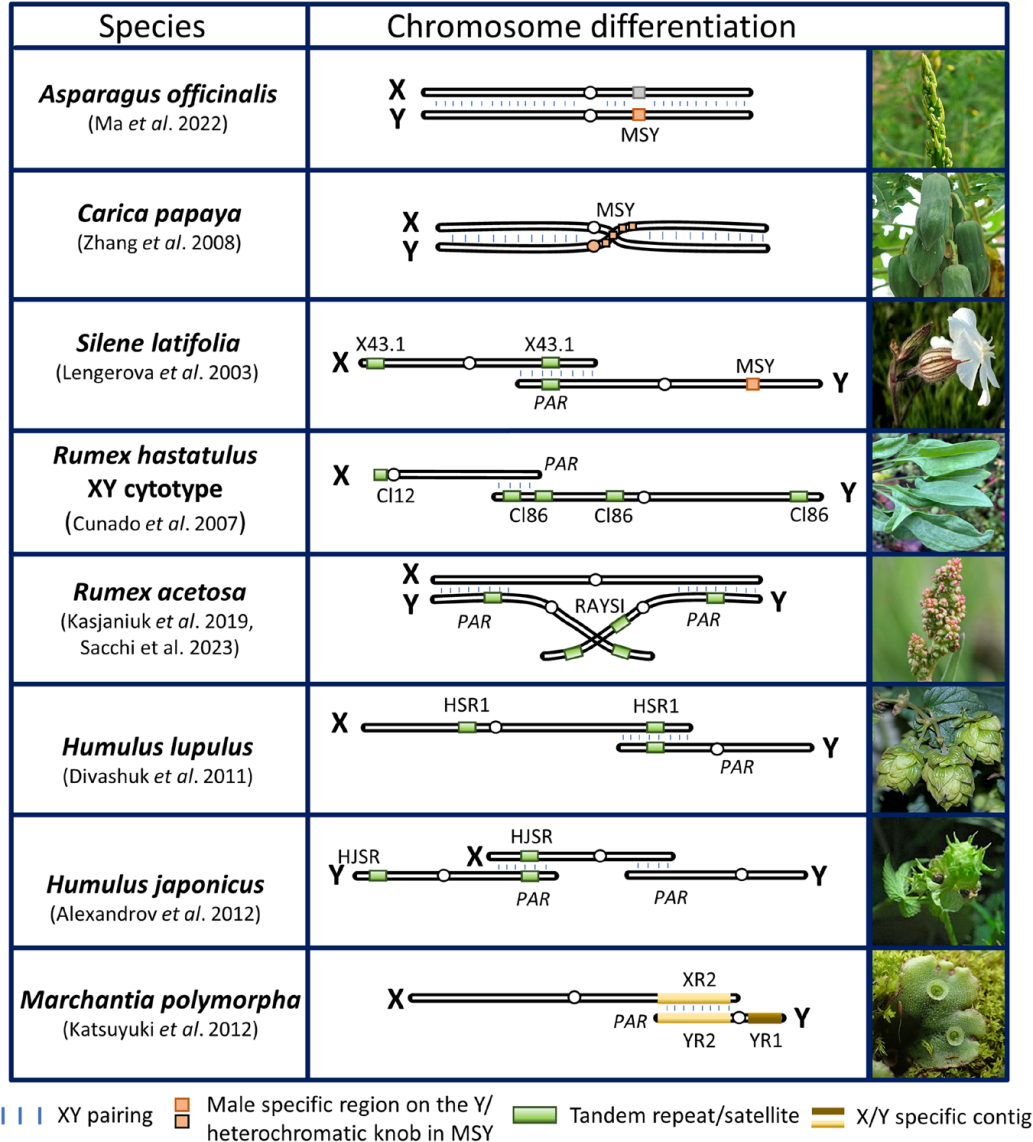
Schematic diagram of sex chromosome evolution in dioecious plants. Species are shown according to their level of sex chromosome differentiation and Y chromosome asynapsis. In *S. oleracea*, *A. officinalis*, and *C. papaya*, the sex chromosomes are mostly homomorphic with recently formed non-recombining regions (region with suppressed recombination). The non-recombining region is largely extended almost to the entire chromosomal length in species with heteromorphic sex chromosomes, namely in *S. latifolia*, *R*. *hastatulus* (XY cytotype), *R. acetosa*, *H. lupulus*, *H. japonicus*, and *M. polymorpha.* The position of the centromere, the PAR length, and the ratio between X and Y is illustrative.

Dioecy represents an extreme strategy of sexual reproduction where sex-specific structures emerge on distinct plants. The existence of different sexes frequently gives rise to what are known as sex chromosomes (typically X and Y or Z and W). The widespread occurrence of recombination suppression within sex chromosomes is a common evolutionary trend, typically accompanied by degeneration and the loss of genes in the non-recombining region of the sex-limited chromosome (Y or W) (Fig. 1). The evolution of the Y(W) chromosome, or Yh in papaya (VanBuren *et al.*, 2015; Yue *et al.*, 2022), involves key stages such as the establishment of the sex-determining region, suppression of recombination, accumulation of repeats, gene degeneration, and reduction through deletions. Expansion and shrinkage are frequently concurrent processes that shape the Y chromosome structure, exerting varying impacts on Y chromosome dynamics throughout different stages of sex chromosome evolution (Vyskot and Hobza, 2015).

The nature and complexity of sex chromosomes often demands cutting-edge technologies for comprehensive analyses of their evolution and correct assembly of non-recombining regions (Fig. 1). Consequently, classical methods in genetics that utilize genetic maps for comparative analyses, genome rearrangement analysis, and gene identification are limited due to the repetitive fraction within the sex chromosomes and suppressed recombination. Even genetic mapping based on deletion mutant lines can be challenging if those deletions are lethal during gametogenesis. The approach used to avoid this limiting factor was for a long time the use of radiation hybrid (RH) or HAPPY mapping approaches (for a review, see Riera-Lizarazu *et al.*, 2008). However, the application of these methods in plants is still in the experimental phase and quite challenging. Currently, the huge progress in methods improving genome assemblies, such as optical genome mapping, makes the study of long non-recombining regions more feasible. This progress opens avenues for deeper exploration, potentially uncovering novel insights into sex chromosome evolution and facilitating more accurate assembly of non-recombining regions. The integration of third-generation sequencing techniques, supported by functional analysis and cytogenetics, not only will enhance our current understanding of sex chromosome origin and the role of chromosomal rearrangements during sex chromosome formation, but also paves the way for future discoveries regarding the non-recombining region and evolutionary strata.

In this review, we aim to highlight some peculiarities of sex chromosome analysis resulting from the aforementioned aspects. The purpose of the review is not to enumerate successful applications of individual methods across all plant species with sex chromosomes but to demonstrate their suitability and utility through specific examples.

## Dissecting sex chromosomes: a swift transition from disorder to understanding

**Fig. 2.**
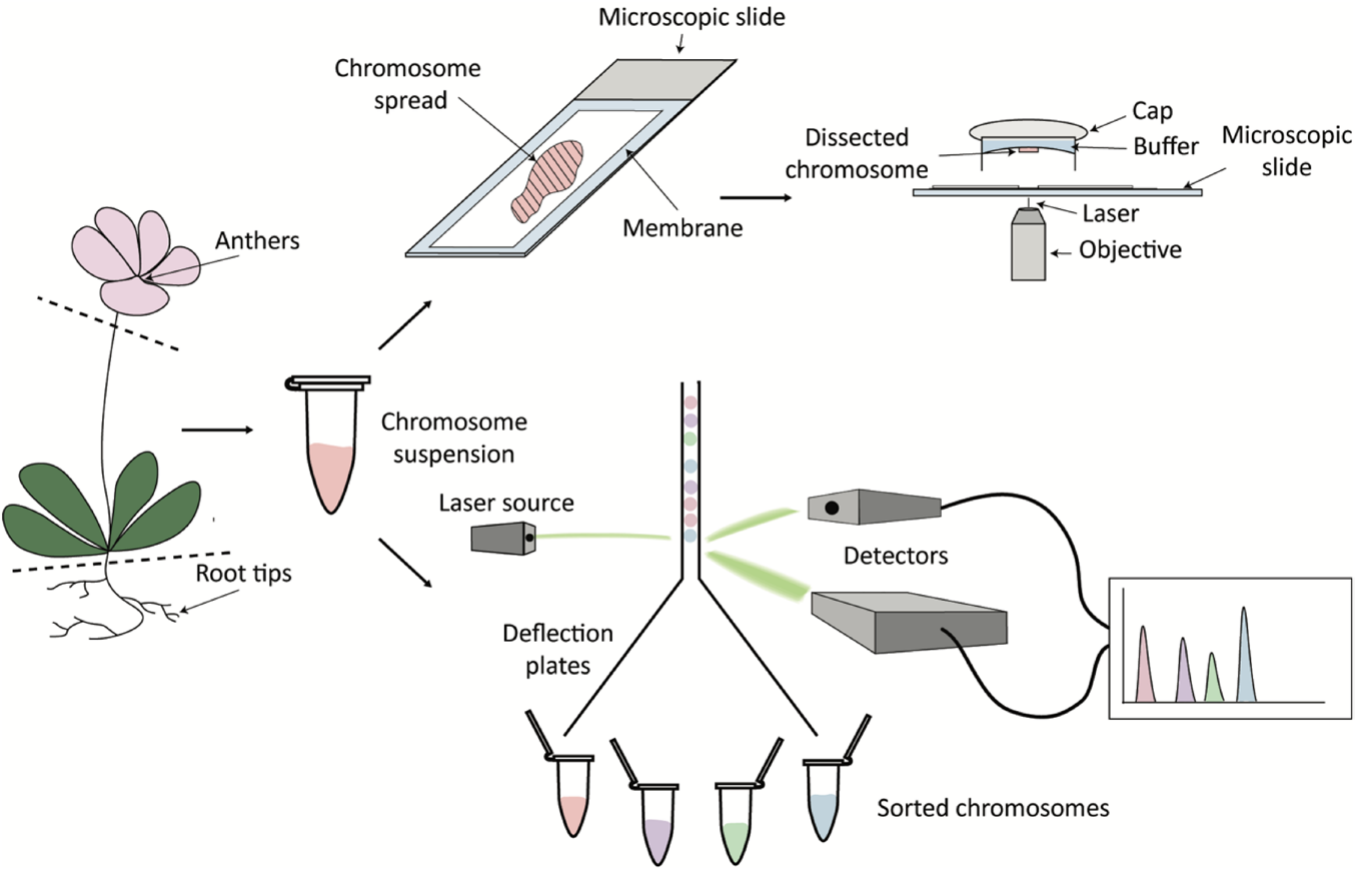
Laser microdissection as a tool to reduce genome complexity. Sex chromosomes in metaphase are isolated from plant cells (mostly pollen mother cells or root tips) and subsequently spread on a special microscopic slide covered with a membrane. After microdissection, chromosomes are transferred into a tube and processed by other applications. In the case of chromosome sorting, the chromosome suspension is stained with a DNA-specific dye and introduced into a flow chamber. Within this chamber, individual chromosomes interact with a laser beam, and the scattered light and emitted fluorescence are measured. Through this process, a histogram of fluorescence intensity (known as a flow karyotype) is generated. Sorting is accomplished by breaking the liquid stream into droplets and electrically charging the droplets containing the chromosomes of interest.

Laser microdissection and chromosome sorting of plant chromosomes represent distinct technology designed to simplify the analysis of large plant genomes by physical separation of their specific parts (Fig. 2). Flow sorting is a method of choice when a large volume of high molecular weight DNA suitable for further detailed analysis is required. Flow sorting is relatively easy to perform and, once adapted (e.g. time or strength of fixative), it usually takes from several hours up to several days. In contrast, chromosome microdissection typically ensures higher purity of isolated chromosomes (almost 100%) and it might be usable for a wider range of applications (Hobza and Vyskot, 2007; Soares *et al.*, 2020). Nevertheless, both manual and laser beam-based microdissection methods provide significantly smaller amounts of material compared with flow sorting technology. Moreover, microdissection-based methods rely heavily on the expertise of the personnel involved, and the collection of plant material may take from several days up to weeks.

In plants, chromosome sorting and microdissection techniques have been extensively applied, particularly in the analysis of sex chromosomes. Indeed, the dissection of the largest chromosome in spinach (*Spinacia oleracea*) and its amplification by degenerate oligonucleotide-primed (DOP)-PCR helped to identify a male-specific marker (T11A) that was isolated from amplified DNA (Onodera *et al.*, 2008). This led to the direct evidence of the Y chromosome. The X/Y chromosomes in spinach were recently assembled using single-chromosome sequencing and the advantage of manual microdissection (Li *et al.*, 2023). In addition to identification of sex-specific markers and sequencing projects, the microdissection of single chromosomes further helped to develop complex chromosome painting probes as in the case of white campion (*Silene latifolia*) (Hobza *et al.*, 2004) and Japanese hop (*Humulus japonicus*) (Yakovin *et al.*, 2014). Alternative applications fulfil diverse objectives, including the targeted development of DNA markers and the construction of DNA libraries (Požárková *et al.*, 2002; Hobza *et al.*, 2006), physical mapping of individual markers and genes (Cegan *et al.*, 2010), gene cloning (Thind *et al.*, 2017), identification of horizontal gene transfer (Talianová *et al.*, 2012), PCR-based mapping of markers on individual chromosomal arms, genome sequencing (Martis *et al.*, 2013), and validation of whole-genome shotgun sequence assemblies (Kreplak *et al.*, 2019).

While the popularity of sex chromosome (laser) microdissection seems to have dwindled nowadays, it is still a powerful tool to address many questions. The efficacy of laser microdissection extends seamlessly to other fields such as transcriptomics and proteomics, where precise tissue separation based on cellular anatomy or morphology is indispensable (reviewed in Misra *et al.*, 2014; Yin *et al.*, 2023). Overall, this method continues to be utilized in genomic analysis and is likely to remain a cost-effective choice for various genomic analysis, especially in non-model organisms with large genomes.

## Cytogenetic tools to study sex-specific traits

**Fig. 3.**
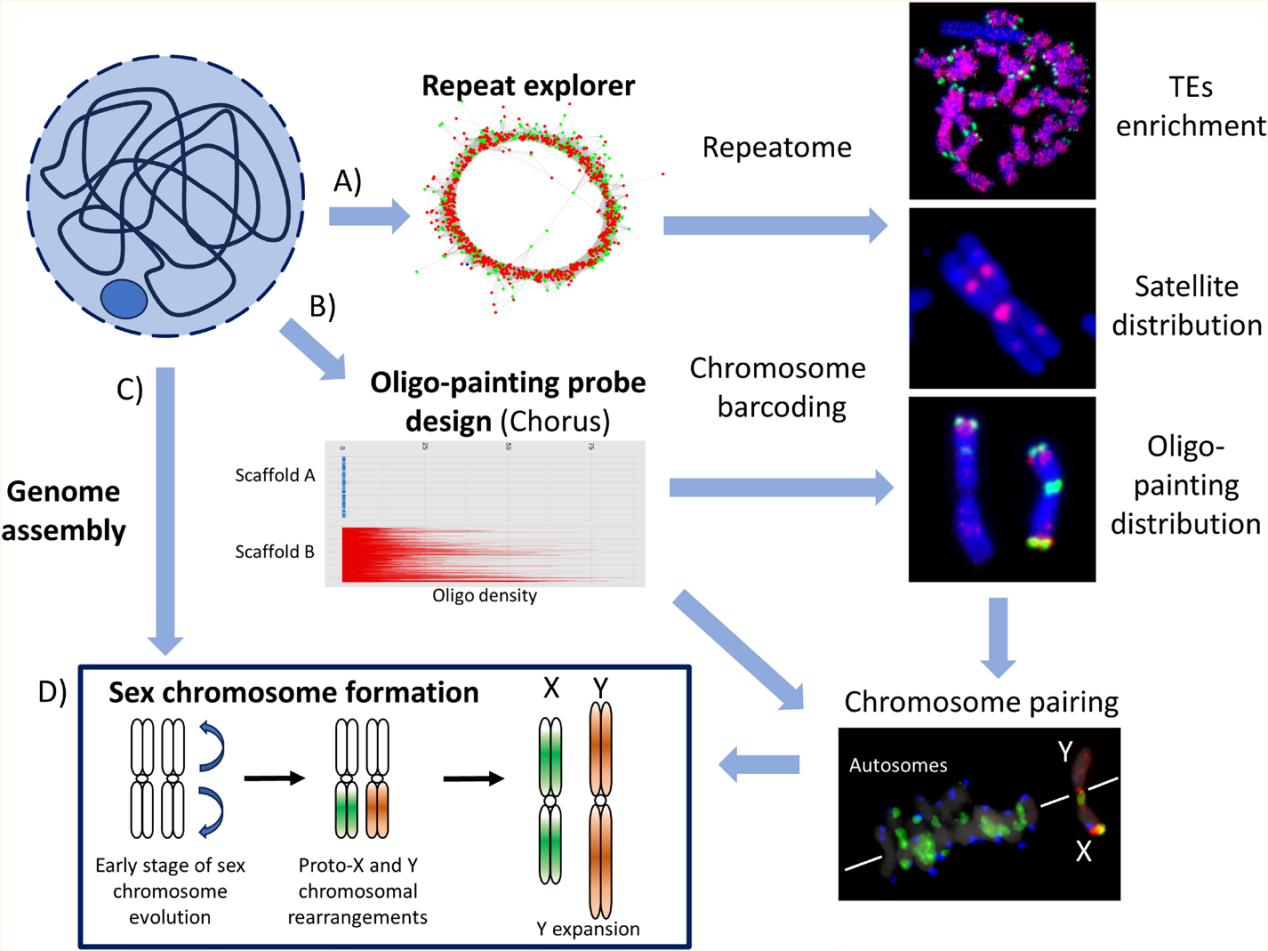
Cytogenetic tools to study sex chromosome origin and evolution. Cytogenetics nowadays combines genomic tools to study repeat fractions including transposable elements (TEs) and satellites (A), to design unique barcodes to distinguish particular chromosome or chromosomal domains using chromosome oligo-painting probe design (B), and bioinformatic tools to dissect single chromosomes or genome parts (C). The combination of the above methods helps to understand sex chromosome evolution regarding their autosomal origin, chromosomal rearrangements, and Y(W) chromosome differentiation. Arrows represent evolutionary steps during sex chromosome divergence (D). The sex chromosome barcoding allows understanding of meiotic pairing which in turn supports chromosomal fusions and inversion/translocations. Chromosomes belong to species with references, from top to bottom, as follows: *S*. *latifolia Ogre* retroelement (Kubat *et al.*, 2014), *R*. *hastatulus* XY cytotype satellite Cl135 (Sacchi *et al.*, 2023, Preprint), *S*. *latifolia* PAR oligo-painting probe with the subtelomeric satellite X43.1 and centromeric satellite STAR-C (Bačovský *et al.*, 2020), and the same DNA probes on chromosomes in metaphase I in *S*. *latifolia* (Bernasconi *et al.*, 2009; Bačovský *et al.*, 2022).

Recent developments in cytogenetic techniques and significant advances in spatial resolution allowed researchers to study various aspects of plant genome architecture. Since Winge’s identification of basic chromosome number in hop (*Humulus lupulus*; Winge, 1923) and Blackburn’s detailed description of sex chromosome in *S. latifolia* (Blackburn, 1923), cytogenetic applications have been fundamental methods for rapid chromosome identification and sex chromosome characterization (Hobza *et al.*, 2018; Charlesworth and Charlesworth, 2020; Muyle *et al.*, 2022). With the combination of high-quality chromosome preparations from a single root tip of a small seedlings or hairy root cell lines (for more details, see Hobza and Vyskot, 2007; Bačovský *et al.*, 2018) and single leaves of living plants (Janousek *et al.*, 2022), it is possible to analyse the karyotype of single plants. In this section, we describe the most used techniques of fluorescent *in situ* hybridization (FISH) and discuss the need for correlation between DNA sequence and molecular data with the structure and organization of plant nuclei (Hobza *et al.*, 2015; Vyskot and Hobza, 2015). FISH is particularly suited to the study of single markers, low copy bacterial artificial chromosomes (BACs), and repetitive sequences, namely (i) transposable elements (TEs) with dispersed genomic distribution and (ii) tandem repeats (satellites) usually occupying isolated genomic loci (Fig. 3).

### The use of transposable elements for monitoring sex chromosome history

The key feature allowing the use of TEs in cytogenetic studies is their uneven distribution on sex chromosomes compared with autosomes (Cermak *et al.*, 2008; Filatov *et al.*, 2009; Steflova *et al.*, 2013; Kralova *et al.*, 2014; Kubat *et al.*, 2014), contrasting with the uniformity in genomes of hermaphroditic species (Cegan *et al.*, 2012; Wicker *et al.*, 2018; Flasch *et al.*, 2019). The likely cause is that TEs are preferentially active in either the male or female lineage as discussed elsewhere (Hobza *et al.*, 2017).  The male-active TEs are therefore accumulating on the Y chromosome while simultaneously being under-represented on the X chromosome, and vice versa for the female-active TEs. This allows TEs to be used (i) to estimate the size of the pseudoautosomal region (PAR), (ii) to determine the boundaries, and (iii) to determine the ages of evolutionary strata arising from the spread of the non-recombining region (Hobza *et al.*, 2015; Vyskot and Hobza, 2015). Early studies based on this principle were limited to a single TE (Filatov *et al.*, 2009) but, by including diverse TE lineages, a more detailed view can be obtained (Cermak *et al.*, 2008; Puterova *et al.*, 2018). This is because TE lineages active at different stages of sex chromosome evolution leave fingerprints (relative insertion densities) from which the history of non-recombining region expansion can be inferred. The conventional approach used to investigate this phenomenon is multicolour FISH simultaneously analysing multiple TE-derived probes. With the increasing availability of new cytological techniques and whole-genome assemblies, the precision of this approach can be expected to increase through a combination of super-resolution microscopy, such as structured illumination microscopy (SIM) and *in silico* analysis. *In silico* analysis requires precise annotation of individual TE lineages and must include assessment of their past transposition activity based on the determination of TE insertion ages (see below). When these conditions are met, TEs, alongside sex-linked genes, can become a powerful tool to study evolution of non-recombining sex chromosomes and to identify cryptic sex-linked regions in homomorphic sex chromosomes.

### Satellite analysis in the context of sex chromosome biology

Repetitive sequences in non-recombining regions of sex chromosomes undergo rapid evolution, diversification, and expansion (Lengerova and Vyskot, 2001; Mariotti *et al.*, 2008; Navajas-Pérez *et al.*, 2009), followed by chromatin changes (Kubat *et al.*, 2008; Steflova *et al.*, 2013; Sacchi *et al.*, 2023, Preprint) and the formation of inactive chromatin regions visible as DAPI banding on the metaphase Y chromosomes (Jesionek *et al.*, 2021). The rapid expansion of repetitive sequences leads to genetic divergence and has far-reaching biological consequences, including the formation of reproductive barriers that further fix genetic differences (Kirkpatrick, 2017; O’Neill and O’Neill, 2018; Hooper *et al.*, 2019). It also allows the use of repeats to reconstruct the evolution of sex chromosomes at the interspecific level and within a species. The established starting point is the identification and characterization of repeats from short genomic reads by specialized bioinformatics tools (e.g. RepeatExplorer, see below) followed by physical mapping using multicolour FISH.

The most suitable candidates for these analyses are satellites creating large arrays in lengths of tens to thousands of kilobases. Among satellites, we also count robust cytogenetic markers of rDNA clusters (45S, 5S). The cytological mapping of 5S rDNA in *Rumex hastatulus* XY and XYY cytotypes helped to identify the autosomal pair that fused to sex chromosomes resulting in the formation of the neo-XYY sex chromosome system (Grabowska-Joachimiak *et al.*, 2015). Additionally, phased assemblies of both *R. hastatulus* cytotypes revealed that neo-sex chromosomes in younger cytotypes (XYY) were formed by two events: an X–autosome fusion and a reciprocal translocation between homologous autosomes and the Y chromosome. These rearrangements were supported by physical localization of eight satellites indicating that the formation of the new cytotype was accompanied by chromosome shattering (Sacchi *et al.*, 2023, Preprint). Hence, both classes of DNA repeats (TEs and satellite clusters) provide fast information about genome reorganization and are valuable in the identification of chromosomal rearrangements during sex chromosome evolution (Fig. 3A).

### Advances of chromosome-specific labeling

The most significant improvement in FISH application is the development of synthetic oligonucleotide probes (Fig. 3B–D) that are specific for chromosomal region(s), chromosomal arm(s), or whole chromosomes (Jiang and Gill, 2006; Jiang, 2019). With the increasing number of high-quality genome assemblies, such oligo painting probes are expected to become an important and essential tool to study genome evolution between related species as shown for *S. latifolia* and identification of XY-orthologous regions in *S. vulgaris* (Bačovský *et al.*, 2020). Recently, the oligo painting probes helped to anchor Y-specific contigs in the genomic context of *S. latifolia* (Akagi *et al.*, 2023, Preprint).

## Epigenomic landscape of sex chromatin

While most epigenetic methods used in the field of sex chromosome biology are adopted from model organisms, the application of these methods often brings surprising and pivotal conclusions regarding the divergence of XY chromosomes. In this regard, the question of whether epigenetic changes are the cause or consequence of the evolution of sex chromosomes and related phenomena, such as dosage compensation, is still unresolved (reviewed in Muyle *et al.*, 2017, 2022).

The term epigenetics represents all heritable and stable changes in gene expression that occur through alterations in chromatin structure and DNA methylation. These alterations are profoundly influenced by various developmental and environmental factors, driving spatio-temporal chromatin dynamics and the overall structure of the epigenomic landscape. We describe recent methodological improvements that increased our knowledge of sex chromosome epigenomics and discuss the possible use of techniques that are being adopted now or will be in the near future in plant research.

### Chromatin structure of plant sex chromosomes

Before the development of advanced next-generation sequencing (NGS) techniques, such as ChIP sequencing (ChIP-seq), methylated DNA immunoprecipitation sequencing (MeDIP-seq), and other NGS-based methods, plant cytogeneticists were among the first researchers who studied sex chromosomes at the chromatin level. Indeed, early cytogenetic findings in *Silene* and *Rumex* revealed phenomena related to late replication of X chromosomes (Siroky *et al.*, 1994, 1999) or formation of Y-chromosome bodies at the cell nucleus periphery in males (Lengerova and Vyskot, 2001; Vyskot and Hobza, 2015). A fresh perspective on immunolocalization involves the use of super-resolution microscopy techniques such as SIM and stimulated emission depletion microscopy (STED), which enable researchers to visualize objects beyond the diffraction limit of light. This innovative approach offers unprecedented clarity and detail, allowing for a deeper understanding of cellular structures and molecular interactions, as was demonstrated for pericentromeric histone modifications and their Y chromosome localization in *Coccinia grandis* (Sousa *et al.*, 2016, 2017). Recent interest in the development of artificial intelligence- (AI) assisted image analysis together with high-content imaging technology further opens up new avenues in the research of various biological related phenomena, for example in meiotic stability of sex chromosomes or tissue sectioning and plant development (Pegoraro and Misteli, 2017; Bitton *et al.*, 2021). Utilization of immunolabelling with high-content imaging remains to be adopted in plant sex chromosome research.

A complex screening of active and repressive histone marks can provide a missing link between early cytogenetic findings (Siroky *et al.*, 1999; Bačovský *et al.*, 2019) and RNA-seq studies (Muyle *et al.*, 2018). Such an approach can be later complemented by the already mentioned NGS technique(s) and its modification(s). Combining sequencing with ChIP-seq with appropriate antibodies against, for example, active histone modifications, allows the deciphering of the evolutionary state of sex-linked genes and their level of epidegeneration. ChIP is a robust method and can be considered an enrichment-based technique like DNA immunoprecipitation (DIP). Following the ChIP protocol, DNA-bound protein is immunoprecipitated using a specific antibody, and the bound DNA is then co-precipitated for further analysis. Additionally, subsequent DNA purification allows either the study of selected genes through quantitative reverse transcription–PCR (qRT–PCR), or an analysis of precipitated DNA using whole-genome sequencing (Rodríguez Lorenzo *et al.*, 2020). Low input and single-cell methods are sometimes required due to limited plant material, for example endosperm studies or single meristematic tissues. A new and versatile method named cleavage under targets and release using nuclease (CUT&RUN) utilizes a new strategy apart from ChIP-seq. CUT&RUN targets micrococcal nuclease (MNase) to binding sites of the protein of interest through specific interactions, allowing it to have a higher signal-to-background noise ratio and analysis of only thousands of cells per sample. The CUT&RUN approach was successfully utilized in Arabidopsis as an alternative and efficient strategy for plant epigenomic studies but remains to be adopted for dioecious plant research (Zheng and Gehring, 2019).

Recently, the entire Y chromosome assembly complemented by bisulfite whole-genome sequencing in *S. latifolia* helped to uncover the DNA methylation levels within the non-recombining region (Akagi *et al.*, 2023, Preprint; Moraga *et al.*, 2023, Preprint). The sodium bisulfite protocol has been widely used as a method for DNA methylation analysis for decades. This chemical deaminates non-methylated cytosines to uracil and leaves methylated cytosines unchanged. Compared with MeDIP, it allows a more accurate mapping and identification of methylation at single-base resolution, as well as determination of the average methylation level in the CG, CHG, and CHH context and the identification of differentially methylated regions (DMRs). The main weakness of bisulfite modification is that it is impossible to discriminate between methyl cytosine and other enzymatic oxidation derivatives (oxi-mCs). Oxi-mCs are present in significant amounts in plants, and specific DNA modification protocols are available for each modification (Plongthongkum *et al.*, 2014; Wang *et al.*, 2015; Kalinka *et al.*, 2023).

The cutting-edge sequencing technology of Oxford Nanopore Technology (ONT), Illumina short reads, and Hi-C led to the fine-tuned genome assembly in a female willow tree (*Salix dunnii*) (He *et al.*, 2021). Third-generation sequencing techniques represented by single molecule real-time sequencing (SMRT) from Pacific BioSciences and nanopore sequencing from ONT provide longer reads than conventional methods, starting from an average length of 10 kb up to ultra-long reads >100 kb. Regardless of the detection specificity, both methods can identify epigenetic changes in the nucleotides without previous enrichment or chemical modification (Chen *et al.*, 2023). Combined with the length of the reads, these methods are becoming a promising tool in sex chromosome epigenetics, including, among others, oxi-mC detection. Nevertheless, the functional role of these derivatives in the context of sex chromosome evolution remains to be elucidated.

Increasing knowledge of epigenetic mechanisms related to sex determination in poplar (Bräutigam *et al.*, 2017), melon (Latrasse *et al.*, 2017), and Japanese persimmon (Akagi *et al.*, 2016) shows the importance of chromatin analysis in studies focused on regulation of female and male development. Although the regulatory hierarchy of histone marks and DNA methylation is still elusive, with the substantial improvements in genomics and cytogenetics it is now possible to assess complex regulatory networks and to study remarkable evolutionary convergence of sex chromosomes.

## Bioinformatics of sex chromosomes: unique tools and approaches

Due to the complexity and unique biology of sex chromosomes, it is necessary to develop and modify traditional bioinformatic tools to account for biological phenomena associated with sex chromosomes, specifically considering the segregation of X and Y (Z and W) chromosomes and the presence of a large region with suppressed recombination. The sequencing of complex regions of the Y(W) chromosome is challenging for assembly and subsequent analyses. This is illustrated by the history of assembling the human Y chromosome (the complete sequence was published recently, Rhie *et al.*, 2023). Indeed, reports on dioecious plant genome assemblies with repeat annotation of sex chromosomes are rather sparse so far. It is becoming increasingly apparent that overcoming these difficulties is a priority task, because detailed annotation of repeats can contribute significantly to understanding the evolution of sex chromosomes, for example the expansion of the Y non-recombining region as discussed above. In addition, sex chromosomes appear to be an excellent model system to study the biology of repeat accumulation and evolution, such as the causes of sex-biased TE activity (Kubat *et al.*, 2014; Hobza *et al.*, 2017) and the evolution of satellites located in different genomic contexts in terms of selection and recombination frequency (Jesionek *et al.*, 2021).

### Detection of repeats in full-length sex chromosome assemblies

The list of approaches utilized for repetitive DNA determination in assemblies of dioecious plant genomes is summarized in Supplementary Table S1. As can be seen for *R. hastatulus* and *Silene* spp., the most recent approaches for repeat detection in full-length plant sex chromosomes are based in paticular on employing the Extensive *de novo* TE Annotator (EDTA; Ou *et al.*, 2019). This tool has the capacity to reveal most of the transposons and their (super)families [long terminal repeat (LTR) retrotransposons; terminal inverted repeats (TIRs); miniature inverted transposable elements (MITEs); and Helitrons]. Nevertheless, when applied for *de novo* identification of TEs without the availability of a species-specific TE library, RepeatModeler2 (wrapped within EDTA) is needed for generation of the corresponding LTR retrotransposon sequence library. The TEs are coarsely designed as Ty1/*Copia*, Ty3/*Gypsy*, and Unknown, requiring manual annotation. Beside the complex EDTA pipeline, the RepeatMasker (Smit *et al.*, 2015) is employed using a TE species-specific library generated either *de novo* with RepeatModeler2 (*Cannabis sativa*, *Hippophae rhamnoides*, *Salix viminalis*, *S. cheanomeloides*, and *S. arbutifolia*; Supplementary Table S1) or using available repeat elements from databases (TIGR Plant Repeat Databases, Ouyang and Buell, 2004; and/or RepBase, Bao *et al.*, 2015; e.g. *Carica papaya*, Supplementary Table S1).

Regardless of the presence of sex chromosomes, annotation of the dominant component of repeats in plant genomes, the LTR retrotransposons, suffers from (i) poor lineage-level annotation (superfamily level only) and their (ii) full-length reconstruction from fragments due to multiple mutual nesting (e.g. Jedlicka *et al.*, 2019). A rather general affiliation into superfamilies and/or unknown category can be fine-tuned using tools for annotation of LTR retrotransposon protein domains as well as Domain based annotation of transposable elements (DANTE; https://github.com/kavonrtep/dante) and/or an LTR retrotransposon classification tool such as Tesorter (Zhang *et al.*, 2022). Even though most studies avoid nested TE analysis due to its complexity, there are some tools utilized for successful assembly. One of them is the TE-greedy-nester (Lexa *et al.*, 2020), which employs an iterative greedy algorithm for reconstruction of full-length TEs. This tool provides the most reliable results in combination with Tesorter and REXdb (Neumann *et al.*, 2019) as presented in TE annotation of *Syzygium* tree genomes (Ouadi *et al.*, 2023).

### Identification of repeats using short reads only: RepeatExplorer employment

Due to the above-mentioned obstacles with sex chromosome assemblies, so far most conducted approaches have started with RepeatExplorer (Novák *et al.*, 2010, 2013) run on low coverage short read sequences (e.g. Puterova *et al.*, 2017, 2018; Jesionek *et al.*, 2021; Sousa *et al.*, 2021). These studies used the convenience of RepeatExplorer for producing consensus sequences for (usually) full satellite monomers, which were in turn used for sex chromosome-specific probe design and subsequent visualization with FISH. Beside the satellites, the clusters of LTR retrotransposon fragments were manually curated and used for reconstruction of partial- or full-length Ty3/*Gypsy* and Ty1/*Copia* retrotransposons (e.g. Puterova *et al.*, 2017, 2018). In summary, the most reliable approach used for repetitive DNA detection is a combination of reference repeat detection and annotation from the assembled genomic and/or short read sequences using a combination of RepeatExplorer, RepeatModeler2, DANTE, and Tesorter with subsequent run of robust pipelines as well as EDTA or RepeatMasker exploiting the convenience of the obtained annotated references libraries.

### Identification of sex-linked genes

Identification of sex-linked genes, sex-determining regions, and sex chromosome-specific sequences can be done using different approaches (Supplementary Table S2) which can be divided into three main groups based on the input data [comparison of the coverage in male and female genomic data, transcriptomic or genomic sequences from defined crosses, and association and single nucleotide polymorphism (SNP)-based methods applied to natural populations]. All the presented tools and their corresponding links, including their pros and cons, are listed in Supplementary Table S2.

#### Genomic approaches

One of the first described approaches used to systematically discover Y chromosome genes was the chromosome quotient (CQ) (Hall *et al.*, 2013). In the CQ method, genomic DNA from males and females is sequenced independently and aligned to candidate reference sequences. The female to male ratio of the number of alignments to a reference sequence is used to determine whether the sequence is Y-linked. Another option can be a k-mer-based approach which was used by Akagi *et al.* (2014, 2018) for identification of the sex-determining region in *Diospyros kaki* (Akagi *et al.*, 2014) and in kiwifruit (Akagi *et al.*, 2018). Briefly, reads from samples of the same gender were pooled and searched for the presence of gender-specific 35 bp k-mers. Reads including male-specific k-mers were assembled to generate Y-linked genomic contigs. Rangavittal *et al.* (2019) introduced a k-mer-based method called DiscoverY, which combines proportion sharing with female reads with depth of coverage from male reads to classify contigs as Y chromosomal. DiscoverY is an effective method to isolate Y-specific contigs from a whole-genome assembly. However, regions homologous to the X chromosome remain difficult to detect. Another recently developed tool is FindZX (Sigeman *et al.*, 2022), an automated Snakemake-based computational pipeline for sex chromosome identification and visualization through differences in genome coverage and heterozygosity between males and females.

#### Transcriptomic/expression-based approaches in controlled crosses

Transcriptomic approaches represent relatively cheap and very efficient tools for the study of sex-determining systems in non-model organisms. Several tools were utilized to identify sex-linked genes and have been adopted for species without a reference genome assembly.

The LINKYX pipeline is based on the utilization of data obtained by transcriptome sequencing of parents and separately pooled male and female progeny (Michalovova *et al.*, 2015). The main aim of this pipeline is to identify putatively sex-linked markers that should be further experimentally tested. In addition to the X- (or Z-linked) linked SNPs that are identified with the LINKYX_X variant of the pipeline, LinkYX enables identification of putative sex-specific genes (Y specific or W specific) based on the quantitative study of the transcription level in parents and in the dataset of pooled male and female progeny (LINKYX_Y). LINKYX_X and LinkYX have been successfully applied to the study of sex determination in *S. otites*, *S. borysthenica*, and *S. colpophylla* (Balounova *et al.*, 2019). SEX-DETector and SEX-DETector++ (Muyle *et al.*, 2016) are, similarly to LINKYX, based on the study of the transcriptomes in the population obtained by a controlled cross. This method has been shown to work well with as few as five offspring of each sex and has been used successfully in several dioecious species (Muyle *et al.*, 2018; Martin *et al.*, 2019; Veltsos, 2019; Badouin *et al.*, 2020; Fruchard *et al.*, 2020; Prentout *et al.*, 2020). SEX-DETector and its updated version can in fact be used for multiple purposes: identification of sex-linked genes and sex chromosomes in the studied organism (XY or ZW), haplotype reconstruction of the gametologue copies, and estimation of allelic expression of each of the copies. However, because of its requirement for a controlled cross, the use of this method is limited to species that can be easily bred or cultivated in controlled conditions. Therefore, this hinders its application to species with a long generation time.

#### Association and SNP-based methods in wild populations

To characterize the sex determination system, genome-wide association studies (GWASs) were used in several species (e.g. *Salix nigra*, Sanderson *et al.*, 2021; *Dioscorea alata*, Mondo *et al.*, 2021; *P. euphratica* and *P. alba*, Yang *et al.*, 2020). As input, the genomic sequences, DarTSeq reads (Diversity array technology), and capture array were used for mapping to reference sequences. For use of restriction site-associated DNA sequencing data to study sex determination, a computational workflow RADSex (Feron *et al.*, 2021) was developed. This tool was developed for Japanese medaka (*Oryza latipes*) (Feron *et al.*, 2021), but it can be adopted in other species, including plants. Sex-specific markers can be further identified with Double digest restriction-site associated DNA sequencing (DdRADseq), a method that was used in *Nepenthes* (Scharmann *et al.*, 2019) and *Amaranthus* (Montgomery *et al.*, 2019). To improve the robustness and transparency in sex-linked sequences identification, Grayson *et al.* (2022, Preprint) prepared a comprehensive workflow called SexFindR. This workflow combines coverage-based analysis and a variety of population genomic analyses such as the reference-based SNP density, GWAS, and F_ST_, as well as the reference-free k-mers GWAS to screen for common candidate sex-linked regions. Sdpop (Käfer *et al.*, 2021) has similar goals to SEX-DETector and SEX-DETector++ but is based on different models and so it enables identification of sex-linked transcripts in natural populations. This approach has been used to study sex chromosomes in *Amborella trichopoda* (Käfer *et al.*, 2022).

New bioinformatics tools and approaches substantially increased the number of dioecious plants identified to date These tools helped to characterize the biological nature of sex chromosomes, leading to important discoveries in epigenetics and functional genomics, enabling the study of sex-linked genes through genome editing. In the last section, we summarize the historical and the most used techniques in the field of functional genetics and discuss future directions.

## Functional genetics of plant sex chromosomes

**Fig. 4.**
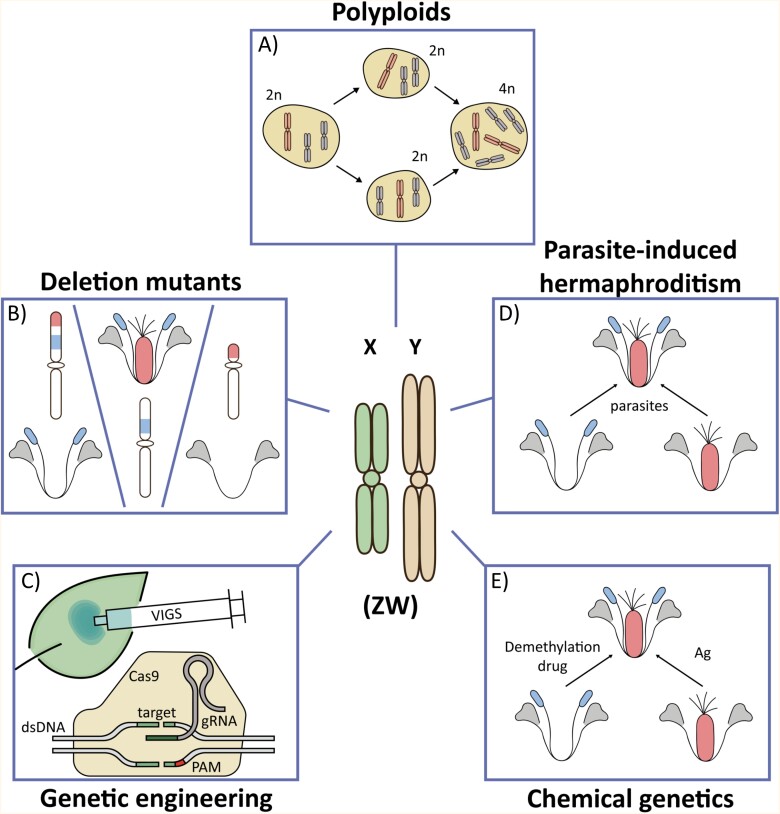
Strategies to assess the function of sex chromosomes in plants. Experimental assays with polyploids (alternatively aneuploids) represent the classical way to determine the role of individual sex chromosomes (A). These assays with plants of various ploidy levels are usually supported by analyses of deletion lines (plants carrying short chromosomal deletions or microdeletions) (B) that allowed researchers to identify sex-linked regions involved in sex determination and floral development. Modern assays using reverse genetics, such as CRISPR/Cas9, virus-induced gene silencing (VIGS), or peptide treatment of shoot apical meristems (C), provide direct evidence of the gene function and its contribution to the development of reproductive organs. Parasite-infected (D) or chemically induced (E) hermaphrodites from either female or male individuals, such as *Silene* or kaki, led to the identification of key mechanisms and genes that regulate sexual phenotypes, and to understanding of the regulatory networks leading to separate sexes.

Sex chromosomes carry the decisive information as to whether the individual will become male or female; however, functional studies of plant sex chromosomes and sex-linked genes are generally not straightforward. While classical genetics rely on recombination mapping for identification of causal genes, this approach is largely unfeasible as sex-linked genes are located within non-recombining regions. Moreover, extensive accumulation of TEs and/or tandem repeats makes genome assembly difficult, which is especially the case for Y/W chromosomes (for details see previous sections). On the other hand, the scientific community developed and applied diverse strategies to overcome the issues related to studying functional aspects of plant sex chromosomes (Fig. 4A–E).

### Classical strategies to study functional aspects of plant sex chromosomes

Although cytogenetic techniques allowed the identification of heteromorphic sex chromosomes in numerous plant species (for a review, see Ming *et al.*, 2011), their role in the sex determination mechanism was not immediately clear. Initially, phenotypic analyses of individuals with numerical chromosomal abnormalities were essential for deciphering the contribution of particular chromosomes to sex determination (Parker and Clark, 1991). Polyploid and aneuploid plants are key materials to elucidate whether sex is determined by an active Y(W) chromosome (as in humans) or by the X:autosome ratio (the system known from *Drosophila*). The active Y(W) chromosome has been observed in the majority of dioecious plants, such as *Carica papaya* (Hofmeyr and van Elden, 1942; Hofmeyr, 1944), *Coccinia grandis* (Kumar and Viseveshwaraiah, 1952), *Silene latifolia* (Ono, 1939; Warmke and Blakeslee, 1939; Westergaard, 1940), *Silene otites* (Warmke, 1942), and *Populus tremula* (Johnsson, 1940, 1942, 1945). Conversely, the X:autosome type that is characterized by no effect of Y(W) on sex determination seems to be infrequent in plants, as it was confirmed only in hop (genus *Humulus*; Neve, 1961; Shephard *et al.*, 2000) and several sorrel species such as *Rumex acetosa* or *Rumex hastatulus* (Ono, 1928; Smith, 1963). Despite the fact that the studies of polyploids and/or aneuploids mostly explored the sex determination systems solely on the broad level of whole chromosomes, this classical methodological approach undoubtedly laid the cornerstone of plant sex chromosome research (Fig. 4A).

Deletion mutants represent another significant methodological step toward understanding the function of plant sex chromosomes (Fig. 4B). In his seminal experiments, Mogens Westergaard obtained *S. latifolia* individuals harbouring Y chromosome deletions (Westergaard, 1946a, b, 1948), some of which resulted in remarkable sexual phenotypes. Plants missing the distal part of the Y p-arm developed hermaphroditic flowers, whereas the absence of the proximal segment of the same arm led to the formation of asexual flowers. Based on these observations, Westergaard defined the gynoecium suppression factor (GSF) and the stamen-promoting factor (SPF). Westergaard’s results provided the first mechanistic evidence that two separate loci are involved in the establishment of dioecy. As such, the so-called ‘two-gene model’ for the evolution of dioecy was proposed based on these findings (Westergaard, 1953; Charlesworth and Charlesworth, 1978), and *S. latifolia* became a textbook example for explaining sex determination in plants.

The analyses of Y-linked deletions became a fundamental approach to studying the sex determination in *S. latifolia* (Donnison *et al.*, 1996; Lardon *et al.*, 1999; Lebel-Hardenack *et al.*, 2002; Zluvova *et al.*, 2007; Fujita *et al.*, 2012; Kazama *et al.*, 2016). The mutations were induced by X-irradiation, γ-irradiation, or heavy ion beam irradiation, all of which create deletions spanning relatively short genomic regions, allowing more precise mapping and marker identification (Fig. 4B). In addition, a thorough characterization of these deletion mutants was carried out including extensive cytogenetic and histological analyses, spatiotemporal gene expression profiling, and electron microscopy (Zluvova *et al.*, 2006; Koizumi *et al.*, 2010). By combining comprehensive phenotyping with physical mapping techniques, the detailed map of the *S. latifolia* Y chromosome was constructed (Kazama *et al.*, 2016) and, recently, comparative genomics using wild-type plants and deletion mutants led to the identification of candidate genes for sex determination (Kazama *et al.*, 2022; Akagi *et al.*, 2023, Preprint; Moraga *et al.*, 2023, Preprint). Deletion mutants are a powerful tool for the identification of sex-determining genes especially in species with small non-recombining regions as described in *Asparagus* (Harkess *et al.*, 2017, 2020).

Interestingly, in some cases it is possible to obtain Y chromosome-linked deletions caused by storage of pollen in inappropriate conditions. This phenomenon has been described in *S. latifolia* as one of the causes of hermaphroditism (gerontogony) (van Nigtevecht, 1966). It is possible to hypothesize that the non-recombining regions can be prone to various kinds of genetic damage. This phenomenon has not yet been studied on a detailed level. There are even no proper data from irradiation experiments as the analyses were always focused on the plants showing aberrant phenotypes in the first generation which leads to a high prevalence of Y deletions in the further studied material, and the frequency of autosomal deletions with recessive phenotype remained unknown.

Some level of phenotypic instability is present in most plant sex-determining systems (Delph and Meagher, 1995). Hermaphrodites originating spontaneously enabled ascertainment of the type of heterogamety in some species (e.g. in *S. dioica*), already at the beginnings of genetics (Shull, 1911). In *S. latifolia*, androhermaphrodites were successfully induced by global genome demethylation and/or inhibiting histone deacetylation (Janousek *et al.*, 1996; Bačovský *et al.*, 2022). Likewise, (imperfect) stamen development can be activated in female plants using silver thiosulfate (Law *et al.*, 2002) and *Microbotryum* infection (Strassburger, 1900; Uchida *et al.*, 2003). A putative female suppressor gene was identified in dioecious buffalo grass (*Bouteloua dactyloides*) using pistil smut- (*Salmacisia buchloëana*) induced androhermaphrodites (Chandra and Huff, 2010). Both andro- and gynohermaphrodites represent remarkable tools for studying sex determination and sex-specific development, because, apart from deletion mutants, they contain complete genomes (Fig. 4D, E). For example, a process of stamen development can be studied in the XX background as well as gynoecium formation in individuals harbouring a Y chromosome (Law *et al.*, 2002; Uchida *et al.*, 2003; Kazama *et al.*, 2005; Zemp *et al.*, 2015; Bačovský *et al.*, 2022) (Fig. 4).

A strategy so far not yet widely exploited for exploring the evolution of sex-determining systems from a functional point of view is the study of interspecific hybrids. The sex-linked genes are often subject to adaptive evolution (Zemp *et al.*, 2018; Muyle *et al.*, 2021) and their change can probably influence the rest of the genome (Zluvova *et al.*, 2021). These changes can be visualized in interspecific hybrids between dioecious species and their hermaphrodite relatives; for example, similar histological phenotypes were observed in some deletion mutants in *S. latifolia* and in the interspecific hybrid between an *S. latifolia* female and *S. viscosa* male (Zluvova *et al.*, 2005). The divergent evolution of the genes related to sexual dimorphism can be observed even in closely related species (Demuth *et al.*, 2014; Baena-Díaz *et al.*, 2019; Liu *et al.*, 2020). The divergent gene evolution in very closely related dioecious species can be revealed on a phenotypic level in subsequent generations of brother×sister mating after interspecific crosses (Winge, 1931). This process overcomes functional redundancy widely present in plant genomes and reveals complex interactions between genes in pathways involved in sex determination and sexual dimorphism. Combined phenotypical, genetic, and genomic analyses of recombinant inbred lines (for a review, see Pollard, 2012) of related dioecious species which so far have not been undertaken could shed new light on the complex evolution of sex chromosomes and the rest of the genomes of dioecious species from both a qualitative and quantitative point of view.

### Reverse genetics tools for investigating the function of sex-linked genes

Identification of sex-linked genes with the aforementioned bioinformatic and NGS methods has opened the door to reverse genetics studies (Fig. 4C). However, none of the dioecious plants with sex chromosomes has become a broadly used model system in molecular biology. Therefore, easily accessible tools for solving complex questions related to sex chromosome function are still lacking. Candidate genes for sex determination have been described in still increasing number of dioecious plants including *Diospyrus* (Akagi *et al.*, 2014, 2016), *Asparagus* (Harkess *et al.*, 2017, 2020), date palm (Torres *et al.*, 2018), poplar (Müller *et al.*, 2020; Xue *et al.*, 2020), willow (Sanderson *et al.*, 2021; Hu *et al.*, 2023), kiwifruit (Akagi *et al.*, 2018, 2019), *Silene* (Kazama *et al.*, 2022; Akagi *et al.*, 2023, Preprint; Moraga *et al.*, 2023, Preprint), and many others. Although CRISPR/Cas9 [clustered regularly interspaced palindromic repeats (CRISPR)/CRISPR-associated protein 9] gene editing represents a very powerful tool in model organisms, the functional evaluation of putative plant sex determination genes has been accomplished so far only in poplar (Müller *et al.*, 2020). Most candidate genes were evaluated by heterologous expression in either Arabidopsis or tobacco (Akagi *et al.*, 2014, 2018, 2019; Kazama *et al.*, 2022). In *S. latifolia*, a combination of virus-induced gene silencing (Akagi *et al.*, 2023, Preprint) and shoot apical meristem treatment with synthetic peptides. Kazama *et al.* (2022) suggested the role of the *CLAVATA3* gene in gynoecium suppression. Interestingly, these studies showed that the divergence of sex chromosomes led to the loss of function in the X-linked copy whereas the Y-copy of *CLAVATA3* remained conserved and fully functional. Though the treatment with synthetic peptides did not lead to complete organ suppression in females, it is a suitable tool for models in which genome engineering is not possible, inefficient, or time-consuming, such as *S. latifolia* (Hudzieczek *et al.*, 2019). With the increasing number of new tools in functional genetics, more biological phenomena associated with plant sex chromosomes such as sexual antagonism, dosage compensation, or sexual dimorphism will be investigated from a functional perspective. Such areas can be examined from a new angle, offering valuable insights into their complex mechanisms and evolution.

## Conclusion

This review highlights methodological approaches that are adjusted, utilized, or entirely developed *de novo* for the purpose of sex chromosome analyses in plants. Investigation of plant sex chromosomes often requires adapting the current methods and optimizing their use for dioecious species as in the case of CRISPR/Cas9 technology mentioned above (Fig. 4C). As more sophisticated genetic engineering tools are still emerging (Capdeville *et al.*, 2023) and are being adapted to non-model species (Lee and Wang, 2023; Yan *et al.*, 2023), we will probably witness extensive functional genetic studies of plant sex chromosomes in the near future. Newly developed NGS techniques and bioinformatic workflows are invaluable for genome comparative analysis, transcription profiling, and epigenomic studies regarding sex chromosome evolution. It can be anticipated that the integration of diverse methods from various disciplines, as recently elucidated in the genus *Cucumis* (Pichot *et al.*, 2022), will provide a more comprehensive understanding of chromatin regulation and sex chromosome compartmentalization within the genome of studied organisms, thereby allowing for a careful rediscovery and revision of important biological questions regarding the origin of sex chromosomes.

## Supplementary data

The following supplementary data are available at *JXB* online.

Table S1. Repeat DNA detection approaches used for dioecious plant genome assemblies.

Table S2. Tools for sex chromosome and sex determination analysis.

erae173_suppl_Supplementary_Tables_S1-S2
